# Impartation

**Published:** 1861-05

**Authors:** W. H. Atkinson


### DENTAL REGISTER OF THE WEST.

VOL. XV.] MAY, 1861. [No. 5.

### Original Essays and Communications.

### IMPARTATION.

(Read before the Mississippi Valley Dental Association, Feb. 20, 1861.)

BY DR. W. H. ATKINSON.

THE limitless variety in the degrees of perception of understanding, as well as of execution, puts it out of the question for the best instructor to make himself apprehended by all the members of his class at once. And as perfection of ideal conception must precede perfection of manipulation, it requires the patient earnestness of goodness to repeat and re-repeat the demonstrations of his curriculum until the last and the slowest in apprehension shall have been illuminated.

What shall I say, then, most to instruct you, my brethren, and thereby secure to myself the illumination of truth? It is not my prime purpose to amuse you; but if I shall be enabled to secure your interested attention, you shall be let into my best fields of idea, thought, opinions, belief and knowledge, which, I hope, may be the means of instructing at least some of us.

Honesty in our inmost is our safest protection, for it is not possible to depart in any degree, and remain free from the evil consequences that will be sure to find us out. Those satisfied to spend their time and money to be merely amused, are the congeners of those pseudo scientifics who are satisfied to take demonstrations at third, fourth or fifth hand, rather than take the pains to pass the ordeals necessary to make them masters of the propositions under consideration. Those willing to take at fifth hand are the class who clearly perceive ideas, and at once miscall them knowledge, and remain incomplete and vague in proportion to the mistake they perpetrate. While those of the fourth hand class correspond to those satisfied with thoughts, only, and foolishly set them down for knowledge, and are ever complaining of the difficulty of making themselves understood; asserting that they see the proposition clearly, but can not tell or write it. Their mistake is like the fifth in kind, but one degree less destructive.

The third class remain in opinions, with the uncomfortable struggle uselessly put forth to reconcile their irreconcilable feelings, until they pass two more ordeals, viz.: belief and well defined knowledge.

The second class approach one degree nearer the culmination of mental effort, and are within one single step of the declarative state, in which they can joyfully say "Eureka," (we have found it.)

Those who persevere are alone the ones properly and legitimately belonging to the first class; who come into relation with the blessed realities of the living embodiment of light, life and truth, (which constitute the trinity of all demonstrations of the departments, as well as grand centers of science): which bring us to the immediate verge of the finite, and compel us to acknowledge the positive certainty of the Infinity above and beyond us. The law of *impartation* is the true means of development, mentally and physically: for the moment we have attained the unfoldment necessary to perception of new truth, it is necessary to "fix it," as it is called, by imparting it to paper, our fellows or to the winds, if neither means of writing nor persons to whom to communicate are at hand.

Were our psychometric powers sufficiently developed, we could imbibe the whole of the products of the mental labors thus written or spoken by simple contact, in the first instance by touch of the book or person, and in the second by the touch of the atmosphere in which the words had been produced—conveying at times to us a fuller and deeper meaning than had found reception in the mental organism that first closed the conjunctive circuit producing the demonstration: fixing the knowledge in our minds far more irradicably than our most approved methods of the former teachers. This *in*-ability for properly dissecting and recomposing mental phenomena has been our chief barrier to certain and satisfactory mental philosophy.

FEELING being the primal exercise of sentients, is necessarily the one ground upon which we can all meet and commune in the true democracy of our natures.

Were all regularly developed from this unitary plain, we should be able at once to assign to each his proper place so accurately as to satisfy all. But an ever-varying impetus relationally presenting itself, hastens one through the entire field, elipsing some and indelibly impressing other incidents of travel upon the recollection, and slowly taking others along, thus some are in one stage of unfoldment and some in another, so that it requires the hand of the master to classify and put them each to his proper work. The primal, claiming to be able to accomplish all that it is possible for any to do,—while the one in advance knows he is not capable the moment he proves him by allowing the test of effort to show him up; so he who will not kindly take the instruction of advanced preceptor must either remain in his low state or be borne out of his errors by the extrusion of failures, coming so sharply to him, as to make him *feel*—imagine—think—opine—believe and *know*, where he does in reality stand. And being thus certainly convicted of his ignorance, errors, and sins, induce him to apply to the only source that ever can *convert* him from the falseness of his ways, and bring him to clear perception of the wholeness of truth.

All may be penetrated through the plain of feeling when caught off their guard that they have put up to keep us out of their inner temple of personal, selfish "sacredness," as they call it. It matters not what may be the particular object or subject of feeling, as soon as it has become epidemic, it carries with it strong and weak alike, if once caught out of this seclusion that annihilates or chokes off *feeling.*

Public opinion is but the concentration of this presence,—that constitutes oneness of feeling, and sways with almost omnipotence all communities of nation, state, country or race—to the accomplishment of a purpose incomprehensible to all save a very few. Hero worship is the great example of this phase of concentrated mental force.

So prolific is the rich mental soil of well fed humans, that every germ dropped into it hastily bursts its pellicle, and urges with tropical ardor the sleeping idea (form type) to a luxuriant and rapid, if not monstrous growth to distortion, in a marvelously short period. The only reason that it is marvelous is because it is new to us, for if we understood the law of development of soul (mental) force in its unitary forms, we could better appreciate it in its aggregations; and should be able to calculate results beforehand in a given case, be it individual or community.

Human beings must have communion—association—and if they have not high and refined forms of society, they as naturally come together upon such terms as present themselves—the unoccupied or negative being most at the mercy of this instinct—feeling. Had we the humility to follow this "instinct"—"first impression," as it is called, we should never make any mistakes. But alas! we begin to calculate, and as we say, "investigate," in whose mazes we soon become lost; and the first moment we lose the thread of our purpose, this mysterious presence (public opinion) impregnates us, and leads us captive, with the tide keeping up a sort of delirium of excitement, until the time and occasion subside, leaving us to the misery of reflections upon misspent time in playing a part we had no real purpose in or appreciative knowledge of.

The man who can see and logically trace conjunctive forces in his earthly mental history only, may well be excused for tarrying in the plain of logical combat. Logic will do as a means, but never as an end or ultimate. But he who recognizes that all he does on earth ought to be the dictate of his guardian spirit or angel, will be less inclined to tarry in the low plain of mere logic, when he may so easily soar into the wholeness of ideal worlds, vivifying all beneath, just as the soul makes and sustains the body.

Bodies may be imperfect representatives of the living principle within, just as our mental natures may fail to express the beauty intended to be symbolized by our guardians of the skies.

Do you ask how is this ? I retort, do you need to be told ? Cast your eyes about you, and show me a perfectly symmetrical body if you can ! But no one doubts for a moment that the germs from which our bodies were developed had an exact degree in perfection, size and proportion, as certainly as that they were not germs of the horse, lion or sheep. Then why are not bodily forms equally perfect as the type by which they were outwrought ? Simply because the physical conditions of development were not as perfect as the type. No builder, be he ever so accomplished, can make a good building out of poor materials. His model may be all that architecture could desire, and his contract in perfect agreement therewith, with him who was to supply the various bricks, mortar and other necessaries; but a failure on the part of this last agent necessarily has its effect on the structure. If the materials are supplied at irregular intervals and in irregular quantities, though of the requisite qualities, a survey of the work will show it to all acquainted with the principles and laws involved. Much more apparent will it be if the quality be also bad. It is not every house that is imperfectly made that the owner throws on the hands of the architect; nay, most houses are in many respects less perfect than the draught called for ; and yet the owner compromises the matter, and moves into and puts up with the imperfections rather than seek to better it in another trial, that might result in no better success. In like manner, our spirits accept our bodies as the best our souls could build for us under the circumstances.

It is pertinent to inquire, what are the circumstances that render us less perfect than the types from which we sprung?

I. Food—constituting the crude materials out of which to construct the parts of our bodily building.

II. The air we breathe, to renovate, purify and desiccate these bricks and cement constituting the walls of our bodies.

III. The company we keep, simulating the workmen who properly or improperly bring together, lay and correlate the parts into one harmonious or discordant whole.

Hence, good food, good air and good company in full play always must produce a harmonic being. And as certainly as this is the happy result under these favorable circumstances, will inharmony and unequal development result in the ratio of departure from good food, air or company. This trinity of circumstances make or mar every human body.

When thus harmonically constructed to its full measure, the body becomes the fit temple for the ghost, or soul and spirit—the soul holding the relation to the spirit that the body does to the soul.

Molecular life, systemic life and conscious life are the trinity that together compose the earthly human life in body.

1st. The sentient or conscious life may be absent, and yet systemic life go on just the same, except in a degree less vivid :—circulation slower—secretions also held somewhat in abeyance, or the reverse of these.

2d. The systemic life may also retire for a time, without permanent injury to the organism, if the molecular life be not retracted at the same time.

3d. Molecular life may in part retire, and yet the being not become disrupted as to original identity. To properly state so as to be apprehended, I will formalize :—

1′. Spirit—corresponds to conscious life.

2′. Soul—to systemic life.

3′. Body—to molecular life.

(*a*) It is very evident that all the purely mental perceptional processes go on in the spiritual plain.

(*b*) Those pertaining to appetites and passions occur in the soul plain exclusively.

(*c*) The molecular actions which constitute integration and resist disintegration are allied to vegetable and mineral life, and properly belong to and occur in body.

1°. Spirit absent—In trance.
Spirit suppressed—In syncope.

2°. Soul absent—In trance and syncope.
Soul depressed or focalized—In ecstacy.

3°. Body absent—When disintegrated.
Body suppressed—In *somatic* death.

Proofs of the above assertions :

1°°. Relations of what the spirit saw in the trance state, when occurring normally and spontaneously, or by anæsthetics and partial syncope.

And the entire absence of all sensation in the body during the sentient lapse.

2°°. Stoppage of the processes of respiration, circulation, secretion and excretion—in trance for hours and days—in syncope for shorter spaces of time.

3°°. The body is absent in just so far as it becomes wasted by emaciation, loss by the disintegration of abscesses, sloughs or mechanical mutilations or chemical changes after somatic death. It is in the suppressed degree in all preparations of whole cadaver or parts charged with antiseptics—this very preservative power owing its efficiency to the infinitessimal division of the life force, here denominated molecular, aggregative, vegetative or mineralogic, and is nearly allied to its fractional expression, denominated solidification.

Just the degree of elasticity or capacity to be elongated pertaining to spirit and soul has not been demonstrated to my vision, but we may safely state that the spirit can not retire to a great distance without also taking its clothing (body) the soul, with it.

The plains of correspondences will throw some light upon this. The spiritual corresponds to the ethereal plains of existences. The soul to the magnetic plain or sphere. And body to crude electrical plain.

Hence, the spiritual will be ethereal—the sympathetic magnetic, and the concentrated electrical merely.

FEEDING has three objects and three modes, the first and second being incipient and introductory stages to the third object with its mode, the focalizing point and culmination or expression for which they were called into play—the construction of habitat for soul and spirit:

1st. To bring together granules to form cells out of whose aggregations spring tissues and organs, to be arranged into system.

2d. Charging these molecules, cells and tissues with magnetic polarity (the apparent inception of sex) by induction, constituting them a fit habitat for the soul proper—so that,

3d. The spirit can inflow and possess the soul, as it had the system, and thus complete the machinery to outwork character in the spiritual sense.

Character, like will, owes its existence to the conjunction of the elements composing it, and is not subject to perception or measurement but by the impress it gives the spirit, soul or body. They all in turn being negative in character when first formed, simulating the paper on which to trace whatever seemeth good to the writer. A certain dominant tendency in these may be present, modifying the ability of character to work in its beautiful designs: just as paper may not always be purely white—dinginess or colors in a degree rendering obscure the impress of the chirographer.

The astute mind will have perceived that a starting point was taken at granules, and may wish to know why I do not go back and expose the inception of molecules, as well as cell and its aggregations.

The reason is plain—to feed implies some *thing* to be fed, and so I began with the granule and fed it to the cell, and so on in the series of elevations and complications of parts until we came to the spirit.

Feeding implies the disruption of old and institution of new relations, from simple imbibition to the most complicated elective affinities in the finest pabulum composed of the richest and choicest ethereal foods.

The chasm is so great between the ethereal spirit of human existences in their present advancement, and the coarse depravity of plant and animal as produced under the destructive influences of what is called civilization, that immediate assimilation is out of the question, and so we are forced to the necessity of long, careful processes of chemical preparations of these for food, or of searching for it in low structures of fishes and other unperverted wild creatures of field and flood, so readily assimilated in their almost living state.

The very fruits even of high culture in some degree having to pass the chemical ordeal to dissipate the falses that had found lodgment in their cells, being shed from the "wicked" or "unwilling" labor that produced them, or collected, transported and sold them: every personal electrosphere they touch either deteriorating or regenerating their qualities for the sustenance of the soul and spirit in the true equipoise of harmonial degrees.

It is said a man is, in character, like what he eats. This elucidation will in some degree suggest the reason why: The digestion of some individuals is so vigorous that it prepares apparently all varieties with equal facility, rejecting non-affiliating elements so promptly as to give them no time nor opportunity to set up schisms in the system. Usually, however, these are examples of mere animals with but very little mental development.

When the individual becomes fine, if fine, he seeks the higher pursuits, leaving the gross drudgery to those of concomitant coarseness, roughness and strength.

We must not here mistake the too prevalent inaction of unwillingness to be alive at all in any useful employ, for this seeking of higher forms of labor; for be it ever remembered that the very law of life is action—equal, steady and persistent—as certainly as that the law of death is the reverse of this, whose inception too often comes to us in the guise of an angel of light, to "*ease*" us of our "*burdens*." O! that we could see that the only safe method of becoming freed of *burdens*, is to carry cheerfully the *burden* to the one proper place, and then will the proper officer remove it from our shoulders, and give us in return the sweet product of earnest, honest, blessed, joyous, cheerful labor!

All legitimate advances to higher artistic or scientific attainment, like natural bodily existence of independent individualized life, must pass the ordeals of parturient throes and the shocks of new and untried atmospheres, that surprise us into explosive, irregular, sometimes discordant notes—shocking to the novice in such matters—but the old, experienced obstetrician congratulates himself and the friends always in proportion to the vigor of the notes of resistance to and expulsion of the new atmosphere; having learned from experience that this very act is the surest means of establishing the new pulmonic circulation, without which no isolated, independent existence could be attained. In the cases enumerated, voice, that constituted individuality, is no objection, but rather matter of gratulation to intelligent listeners. Let, then, these truths not be rejected because of their individuality, but be nurtured with care and affection, and see if they do not grow to something worthy of the notice of the good and the great.

If there be no vigorous resistance to this new professional atmosphere, I shall feel that I have mistaken the time necessary to full gestation in our body, and shall have to adjourn the case, as has often occurred to me in my actual practice in the obstetric field of labor, and still allow the embryon to retain its maternal connections, till the pulmonics shall have attained the requisite perfection to fully act, and thus free the circulation from its placental tortuous windings, by embracing the pure atmosphere in which the uncontaminated life force awaits the opportunity to inflow and constitute the individual a true representative of all that consists in wholeness of being. But if I shall have the happiness to find at this, as at other communings, that some have come to full term, and only await the closing of the circuit to come fully into a vigorous and advanced life, I shall feel that the patient gathering up and aggregating of professional molecules shall not have proved a useless work, though it be the product of odd moments stolen from couch or office hours, at irregular intervals, through a long and arduous experience—which I would rather have heartily endorsed or vigorously rejected, as proof of the earnestness of those who have patience to hear, and thus afford an opening into which to insert the wedge of imparting the essentials that will certainly develop the truth of the matter, under the close scrutiny of sharp inquisition of direct questions and answers.

If propositions are not clear, it is certain that they are erroneous or misunderstood. Objections should not be ruled out, but allowed their full weight, so that enunciator or objector may be set right.

I ask pardon for the last digression—but you will please attribute it to my earnest impetuosity and almost mad desire that you all may see the truth at the same vista at which I have been so fortunate as to apprehend it. I am not willing to close until I have further detailed the phases of digestion, in order to present to the organs the pabulum proper for their support and refinement.

The digestion of *air* is the highest and purest direct method of imbibing life forces. *Air* is a calorifient food we must be supplied with at the shortest intervals; being so easily changed, we deprive it of the life in less time than other calorifients, that hold the life principle with more tenacity (the hydrocarbons for example.)

The digestion of the mouth is uncomplicated and fine—next in grade to inspiration of air—and takes up direct the entities set free by the death and disruption of the organism, capable of being instantly killed and dissolved by the oral fluids.

Digestion in stomach is a grade coarser, and requires a longer time to perform the process.

Jejunal, illeal, colic and rectal digesting being grades lower still in the order enumerated, and capable of supplying the system in the exact ratio of the fineness and activity of the function.

*Perfect digestion* assimilates all, leaving no effete substance to be thrown off. Hence, the finest feeders and the closest digesters are of necessity freest from burdens of effete, offensive matters.

And by the way, that word effete is to the unfolded and illuminated understanding an epitome of all that can properly constitute the most complicated foods and their due preparation to effect the separation of vital and effete portions.

Effete—without living center—literally without embryon or fœtus, or without a quickened germ that can be taken out and appropriated to our uses.

All effete portions of our ingesta must be rejected from our systems, or they will occupy to undue distension the receptacles of food, thus very materially interfering with the processes of nutrition. This is so in a moral, professional and bodily point of view; and I hope you will make the application to the whole range of the three, as time would fail us to go into the detail.

And now for the best methods to get rid of the effete matters we have been forced to take into our moral and professional, as well as bodily stomachs; we will do well to copy nature in the unperverted creatures, and imitate the owl and the deer, and reject these by an inversion of the peristaltic movements of the alimentary canal.

The owl swallows his prey, bird, mouse or other small animal, and after dissolving off all the soft parts, throws up the skeleton upon an old log or stone, in a snug little pile of bones, the only part that is not assimilable, and thus avoids the rupture of his alimentary canal, consequent upon the attempt to pass them on through its tortuous and delicate windings.

The deer eats freely of the wild red plum, swallowing skin, pulp, pit and all, but when he comes to ruminating, he very wisely and quietly, as each cud brings up a pit or two with other matters, ejects them with the accuracy of an expert, comminuting and insalivating the balance, and passes it on to be disposed of in making a nice quality of real venison.

These facts I personally learned by my observations as naturalist when a mere boy, in the wilds of a new country, where neighbors were few and far between.

There are, however, many substances that are properly effete, that are not so readily and safely disposed of. Among them, not unfrequently, are to be found those denominated poisons. These may be present in our food, that announce themselves by destructive effects, the first notice we have of them. Again, slight chemical changes often render an otherwise proper aliment decidedly poisonous. The way best to get rid of them will in some degree be indicated by their character, to *know* which is of the first importance. Many, like the bones and plum pits, may be ejected by inverted action of the " primæ " " viæ." But the only method of escaping the damaging presence of others, is, to hurry them through by increased activity of the main passages, thus by profluvia washing them away at the expense of a heavy draft on our nerve power and the pabulum we had hoped would strengthen us for further and higher activities in the specific field of our operations. Here, as elsewhere, we, alas! do not perceive the evil until it has made quite considerable progress,—the main reason of which is, that we sacrifice simplicity and safety to vanity and fashion, recklessly running the risk of a rapid or more lingering destruction, or a sort of compromise between health and death, and thus become invalids for the balance of our time.

I must say that we who are but invalids, morally and professionally, far exceed in numerical strength our pure, healthy, simple and successful brethren, who never took in falses from which they had to be purged.

And now how to attain this wholesome and efficient status as individuals and as a body is, to us, a question of the utmost moment to have clearly and positively solved.

This question of status is, like the cases we have presented to us to remedy, very complicated, and many times far gone on the road to destruction, which renders the matter doubly difficult to successfully treat; want of *proper development* and *perversion* standing equally in the way of true progress whenever present in the case to be dealt with.

To pursue the symbol of food—Undeveloped tastes or perverted tastes are no safe guide in the selection of proper, or even the most desirable aliments. Tastes so often conform to the conditions under which they are developed as to acquire specific modes, rather than generic ways of displaying their choice. And hence the necessity of "*experimenting*" even here at the very threshhold of our individual existence.

The way to do this in the shortest time and to a sufficient extent is, to compare experiences of all with whom we come in communion. Here we are in danger of becoming "*perverted*," if we depart from our highest feeling of purity, simplicity and truthfulness, irrespective of the apparent delight with which certain tastes are advocated. Follow no lead that your inmost does not fully approve. But here let me say, some discernment is required; for often learners become confused, and call it (the confusion) faith in the leader, and so are led captive, until their confusion had subsided, taking away the false faith it had engendered, leaves them less fit for advancement than before the perversion. Do you ask, how, then, shall we become again fit to advance? I answer, by simply falling back upon first principles, and not blindly move in the dark, again to find we had, instead of going forward, moved in a wrong direction, involving waste of time and energy.

Amusement will do to arrest attention or give relaxation to the overwrought organism which the mind works with.

The acquisition of money can only satisfy natures devoid of the moral perceptions, or those who have smothered these by an arrant selfishness ; for all true good is alike, and must, like knowledge, be distributed, to accomplish the highest results intended.

To accept demonstrations or statements not comprehended is a necessity to the novice—that they may prove stepping stones to the acquisition of real knowledge. But not to *use* them as such, renders us fossiliferous, and inevitably prevents expansion of power.

Whenever you think you have made an advance, be careful to communicate it, that it may be proved, and when proved, distributed to the world, that you may be free to work again, alone or associated.

Most of discovery, thus far, has been the work of isolation ; but the advances most *apparent* are results of association.

Thus we work alone in the acquisition of single items of knowledge, and when we come together, each contributes the particular specimen he has come in possession of, and so we have in the aggregate a very fine cabinet that approximates completeness. In a fraction of the time we could each at home collect and correlate one apiece.

Now, if each member of this oldest dental society will but earnestly put himself to work, and follow the course of true living here indicated, we shall soon have the happiness to know that we are not only the oldest, but the most useful, because most advanced in the associations of our loved and honored professional body.

